# Peripherin: A Novel Early Diagnostic and Prognostic Plasmatic Biomarker in Amyotrophic Lateral Sclerosis

**DOI:** 10.1111/ene.70241

**Published:** 2025-06-06

**Authors:** Alessandro Bombaci, Giovanni De Marco, Federico Casale, Paolina Salamone, Giulia Marchese, Giuseppe Fuda, Andrea Calvo, Adriano Chiò

**Affiliations:** ^1^ Turin ALS Centre, “Rita Levi Montalcini” Department of Neuroscience University of Torino Turin Italy; ^2^ IRCSS Policlinico San Donato San Donato, Milanese Italy; ^3^ Vita‐Salute San Raffaele University Milan Italy; ^4^ SC Neurologia 1U AOU Città Della Salute e Della Scienza di Torino Turin Italy

**Keywords:** fluid‐biomarker, HSP, mimics, neurodegeneration, neurofilament, PLS, PRPH, regeneration

## Abstract

**Background:**

Motor neuron diseases (MND) are heterogeneous and complex neurodegenerative disorders. Biomarkers could facilitate early diagnosis, prognosis determination, and patient stratification. Among the most studied biomarkers are neurofilaments, with peripherin (PRPH), a specific type predominantly expressed in the peripheral nervous system, gaining attention. To date, no studies have evaluated PRPH in human plasma.

**Methods:**

Sandwich‐ELISA was used to quantify plasma peripherin from 120 MND (100 ALS, 4 PMA, 15 PLS), 73 MND‐mimics, and 38 healthy‐controls (HCs). Plasma was collected at diagnosis or some months earlier. 41 ALS were evaluated longitudinally. ALSFRSr, MRC, spirometry, genetic tests, disease progression rate (PR), blood examinations, and neuropsychological tests were performed. Statistical analyses included Kruskal–Wallis, Mann–Whitney, Cox regression, and Kaplan–Meier curves.

**Results:**

Plasma PRPH levels differed significantly among groups (*p* < 0.0001), showing higher values in MND participants than MND mimics and HCs. Moreover, PRPH levels were elevated in PLS compared with HSP patients (*p* = 0.0001). Differences persisted after adjusting for age and sex. ROC curve demonstrated that PRPH discriminated MND from MND mimics (AUC = 0.85). Elevated PRPH correlated positively with ALSFRSr and lower motor neuron index, whereas inversely with disease progression rate. Higher PRPH levels at the beginning of the disease were associated with longer survival.

**Discussion:**

Plasma PRPH is raised in MND, particularly ALS, from the earliest stages, distinguishing MND from mimics and correlating with clinical parameters and survival. This suggests PRPH may reflect an endogenous response of lower motor neuron to injury. Further multicenter studies are required to refine the diagnostic and prognostic utility of PRPH in MND.

## Graphical abstract

Plasma peripherin (PRPH) levels are significantly elevated in patients with motor neuron diseases (MND), including ALS, compared to both MND mimics and healthy controls. Higher baseline PRPH correlates with better functional status, slower disease progression, and longer survival, suggesting it may reflect an endogenous motor neuron injury response. These findings support PRPH as a promising early diagnostic and prognostic blood biomarker in MND, warranting further multicentre validation.

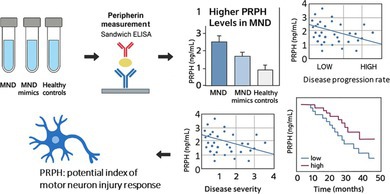

## 1 Introduction

Motor neuron diseases (MNDs) encompass a group of fatal neurodegenerative disorders marked by progressive motor neuron degeneration [1]. Amyotrophic lateral sclerosis (ALS) is the commonest form, involving both upper and lower motor neurons and causing bulbar, limb, truncal, and respiratory weakness [2]. Approximately 10% of ALS cases are familial (fALS); the remainder 90% are sporadic (sALS) [3]. Cognitive or behavioral impairments occur in half, and around 10% progress to frontotemporal dementia (FTD) [1]. Despite extensive research, the aetiology and pathophysiology of ALS remain unclear, with a median survival of about 3 years post‐diagnosis. Other MNDs include primary lateral sclerosis (PLS), involving upper motor neurons, and progressive muscular atrophy (PMA), which primarily affects lower motor neurons.

Reliable biomarkers are required for early diagnosis, prognostication, patient stratification, and trial enrichment. Neurofilaments (NFs) have emerged as promising biomarkers for ALS and other neurodegenerative diseases [4, 5]. Beyond the widely studied neurofilament triplet proteins (NFTPs, i.e., Neurofilament Light Chain [NfL], Neurofilament Medium Chain [NfM], and phosphorylated‐Neurofilament Heavy Chain [pNfH]), and alpha‐internexin, another type III intermediate filament belongs to the group of neurofilaments, the peripherin (PRPH). It forms heteromers as a fourth subunit in the peripheral nervous system's neurofilaments [6]. As well as all the other NFTPs, PRPH presents a stoichiometry fundamental for adequate axonal transport and for appropriate cell structure [7]. Alterations to this stoichiometric balance of intermediate filaments are associated with the formation of cytoplasmic inclusions in motor neurons of ALS models [8]. This phenomenon seems to be the result of the loss of proper regulation of intermediate filament mRNA metabolism [7, 9]. Moreover, PRPH is fundamental not only in axonal transport and constitution of the neuron cytoskeleton, but it also contributes to axonal growth and neuronal differentiation [10].

Serum and cerebrospinal fluid studies have shown raised PRPH in ALS versus dementia, spinal bulbar muscular atrophy, polyneuropathies [11], and in early Guillain–Barré syndrome [12]. Plasma PRPH, however, remains unexplored.

Given the pivotal role of PRPH in motor neuron health and response to stress, this study investigates PRPH levels in the plasma of MND patients. We aim to elucidate its diagnostic and prognostic value, potentially enhancing our ability to differentiate ALS from other neurodegenerative and neuromuscular conditions.

## 2 Materials and Methods

### 2.1 Design of the Study and Clinical Assessment

First, we set up a preliminary test to validate the Human Peripherin (PRPH) ELISA Kit (Abbexa, Cambridge, UK) for measuring PRPH in human biofluids and determining the optimal dilution factor for ALS patients.

Second, we realised a retrospective, longitudinal study on a cohort of 192 patients and 38 healthy controls (HCs) referring to the Department of Neurosciences of the University of Turin (Italy) from June 2019 to March 2023 who underwent blood collection for diagnostic purposes. Blood samples were collected from MND patients at diagnosis and, for 41 ALS patients, again after 6 months. Participants with active cancer, infections, or autoimmune disorders were excluded.

Demographic and clinical data are summarized in Table 3, ensuring robust patient characterization and comparability.

ALS patients received their diagnosis following the Gold Coast criteria [13]. Disease severity was assessed using the ALS Functional Rating Scale‐revised (ALSFRSr) [14]. Subscores included: ALSFRSr_noresp (excluding respiratory items), ALSFRSr_B (bulbar score: items 1 and 3), and ALSFRSr_4limb (limb score: items 4–9).

Moreover, all ALS patients underwent: a clinical evaluation (neurological examination, including the Medical Research Council scale [MRC] [15] measurement of shoulder abductors, elbow flexors and extensor, wrist flexors and extensors, finger flexors and extensors, thumb opposition, hip flexors, Knee flexors and extensors, and foot flexors and extensors, and ALSFRSr scale) at the moment of diagnosis, at the moment of blood sampling (T0), and after 6 months from sampling (T6), an instrumental evaluation (electromyography, cerebral MRI with tractography and functional‐MRI, spirometry), a neuropsychological evaluation [16], and a genetic test for the most common mutation (*C9orf72, SOD1, TARDBP* and *FUS* genes).

Patients were categorized by phenotype (classic ALS, bulbar ALS, respiratory ALS, flail arm, flail leg, and predominant upper motor neuron ALS) [2]. Disease progression rate (PR) and progression rate without respiratory items (PR_noresp) at T0 and at T6 were calculated as follows:

$$\mathrm{PR}\_T0=\frac{48-{\text{ALSFRSr}}_{T0}}{\text{Date}\;T0-\text{Date symptom onset}}$$

$$\mathrm{PR}\_\text{noresp}\_T0=\frac{36-\text{ALSFRSr}\_{\text{noresp}}_{T0}}{\text{Date}\;T0-\text{Date symptom onset}}$$

$$\mathrm{PR}\_T6=\frac{{\text{ALSFRSr}}_{T0}-{\text{ALSFRSr}}_{T6}}{\text{Date}\;T6-\text{Date}\;T0}$$

$$\mathrm{PR}\_\text{noresp}\_T6=\frac{\text{ALSFRSr}\_{\text{noresp}}_{T0}-\text{ALSFRSr}\_{\text{noresp}}_{T6}}{\text{Date}\;T6-\text{Date}\;T0}$$

Moreover, we calculated in ALS patients an index of upper motor neuron (UMN) impairment, called UMN burden score (UMNBS), ranging from 0 to 24, collecting retrospectively data on burden and atrophy distribution from medical records of our Centre (Table 1). This is a readaptation of the Penn Upper Motor Neuron Score (PUMNS) [17], based on data availability. We rated limb spasticity using the Modified Ashworth Scale (MAS) [18].

We also calculated a lower motor neuron index (LMNI; Table 2), implementing the scoring system proposed by Devine et al. [19], whose score ranges between 0 and 12 points, and adding the bulbar lower motor neuron score [20], whose score ranges between 0 and 3 points. The resulting LMNI consists of a complete scale able to evaluate the lower motor neuron involvement both in bulbar and in limb regions, scoring between 0 and 15 points.

Stage of disease was assessed using the two most known systems: the KINGS [21] and the MiToS [22].

**TABLE 1:** Upper Motor Neuron Burden Score (UMNBS).

Limb reflexes (bicipital, brachioradialis, patellar, ankle)	0 if absent, reduced or normal in a normotrophic area^a^ 1 if brisk or retained in an atrophic area^a^
Pathological reflexes	
Palmomental reflex (bilateral), Hoffmann's sign (bilateral), Babinski's sign (bilateral)	0 if absent^a^ 1 if present^a^
Jaw jerk	0 if absent^a^ 1 if present^a^ 2 if brisk^a^
Spasticity at four limbs (as PUMNS)	0 if normal muscle tone 1 if MAS 2–3^a^ 2 if MAS 4–5^a^

^a^ As stated by Woo JH et al. [17].

**TABLE 2:** —Lower motor neuron index (LMNI).

	Lower motor neuron signs
*Limb Score (for each limb) [0–12]*	
0	No clinically significant involvement
1	Definite, but trace involvement Weakness ≥ 4/5, involving one or more segments (with no segments < 4/5), and mild wasting
2	Moderate involvement Weakness ≥ 3/5, involving one or more segments (with no segment < 3/5), and moderate wasting
3	Significant and severe involvement Little or no movement (LMN weakness ≤ 2/5) involving one or more segments, and severe wasting
*Bulbar Score [0–3]*	
0	No clinically significant involvement
1	Tongue atrophy and/or fasciculations
2	Score (1) + tongue hypomobility
3	Score (2) + jaw and/or face weakness

### 2.2 Laboratory Evaluation

Plasma samples for preliminary kit evaluation were collected in EDTA tubes, centrifuged at 1500*g* for 15 min at 18°C within 1 h of collection, and stored at −80°C. Samples were anonymised using unique codes accessible only to authorised researchers. Before ELISA testing, samples were thawed at 4°C overnight, brought to room temperature for 30 min, and centrifuged at 3000*g* for 15 min to remove platelets and cellular debris, following the protocol previously described [23].

#### 2.2.1 Assay

We used the Human Peripherin ELISA commercial Kit (Abbexa, Cambridge, UK), following the manufacturer's instructions.

Each plate contained calibrators (0.312–20 ng/mL) in duplicate and two samples with a known concentration of PRPH, provided in each kit. The manufacturer declared a sample recovery range after spiking of 91%–105% and a linearity range of 90%–116% in dilutions up to 1:8 both in serum and in plasma.

After preliminary analysis, useful for validation of this kit in our samples and led to following protocols adapted from Andreasson et al. [24], all samples were distributed on the plate using a dilution of 1:2 in 0.01 mmol/L of phosphate‐buffered saline (as per kit recommendations), and measured in duplicate. The inter‐assay and intra‐assay coefficients of variance were all below 10%. According to the manufacturer's instructions, analytical sensitivity was set < 0.156 ng/mL.

Plates were read using a CLARIOstar Plus plate reader (BMG LABTECH, Ortenberg, Germany), and standard curves were fitted using 4‐parameter logistic regression with MARS data analysis software. The median intra‐assay and inter‐assay coefficients of variation were below 15% for all the assays.

General blood tests were performed by our hospital laboratory.

### 2.3 Statistical Analysis

A Shapiro–Wilk test showed that the data were not normally distributed.

Kruskal–Wallis and Mann–Whitney tests were used to compare the groups, with Bonferroni post hoc adjustments applied when significant differences emerged. The correlations between PRPH levels and clinical, laboratory, and instrumental parameters were calculated by the Spearman rank correlation (*r*_*s*_). Multiple regression analyses were conducted.

Kaplan Meier curves were generated in patients who underwent death or tracheostomy, also stratifying for age, sex, and site of onset. Multivariate Cox‐regression analysis with a backward stepwise method was led.

Wilcoxon signed‐rank test was assessed for longitudinal analysis.

Statistical significance was set at *p* < 0.05, and all calculations were performed using SPSS Statistics V29 (Chicago, IL, USA). Ethical approval was granted by the Turin ALS Centre's Ethics Committee (Comitato Etico Azienda Ospedaliero‐Universitaria Città della Salute e della Scienza, Torino) (n° 0011613, 03/02/2020), and all participants provided written informed consent.

## 3 Results

### 3.1 Preliminary Evaluations in Biofluids

Using the Human PRPH ELISA Kit, we tested various dilutions (from undiluted to 1:8) of CSF and plasma from 3 MND patients and 3 HCs. PRPH was undetectable in CSF due to levels below the assay's detection threshold. However, consistent with PRPH's peripheral nervous system distribution [6], it was measurable in plasma, with the optimal dilution determined to be 1:2 or undiluted.

### 3.2 Evaluation of Plasma PRPH in MNS Patients, MND‐Mimics, and Healthy Controls

We conducted a retrospective, longitudinal investigation involving 119 patients living with MND (100 ALS, 15 PLS, 4 PMA), 73 cases of MND‐mimics (including myelopathies, radiculopathies, axonal neuropathies, inclusion body myositis, post‐poliomyelitis syndrome, myasthenia gravis with bulbar onset, primary progressive aphasia, Parsonage‐Turner disease, Hirayama disease, syringomyelia, progressive supranuclear palsy, benign fasciculation syndrome, functional disorder, and hereditary spastic paraplegia [HSP]; Table S4), and 38 HCs. Among the MND mimic cohort, 13 patients were found to carry genetic mutations: eight in the SPAST gene, two in the ALT1 gene, one in the ALDH18A1 gene, one in the SPTAN1 gene, and one in the KIF5A gene.

Among ALS, 60 were male (58%), 36 had a bulbar onset of disease (35%), and 2 had a respiratory onset (2%). Among ALS patients, 22 (22%) had a genetic mutation in one of the four major genes: 8 had a mutation in *C9orf72*, 3 in TARDBP, 4 in *FUS*, and 7 in *SOD1*. 7 MND had a concomitant FTD profile. No differences in sex and age between MND, MND mimics, and HCs were observed (*p* > 0.05).

Demographic and clinical characteristics of the participants are summarized in Table 3.

**TABLE 3:** Participant demographic and clinical information.

	ALS (100)	MND mimics (73)	HCs (38)	*p*
Sex (M/F)	60/40	44/29	20/18	0.93^a^
Age at T0 ± SD (y)	64.5 ± 11.9	62.4 ± 13.9	64.2 ± 9.3	0.27^a^
Presence of genetic mutation	21	13	—	—
ALSFRSr_tot at diagnosis ± SD	38.6 ± 5.8	—	—	—
PR at diagnosis ± SD	0.86 ± 0.74	—	—	—
KINGS stage at diagnosis (0/1/2/3/4)	0/27/25/44/4	—	—	—
MiToS stage at diagnosis (0/1/2/3/4)	78/14/8/0/0	—	—	—
FVC ± SD^b^	89.9 ± 20.8^b^	—	—	—
Diagnostic delay (months)	11.2 ± 7.4	—	—	—

Abbreviations: ALSFRSr_tot, ALS Functional Rating Scale‐revised total score; FVC, Forced Vital Capacity; n/a, not assessed; SD, Standard Deviation.

^a^ *χ* ^*2*^ test.

^b^ FVC values obtained around the data of blood sampling available in 74 ALS patients.

**FIGURE 1:**
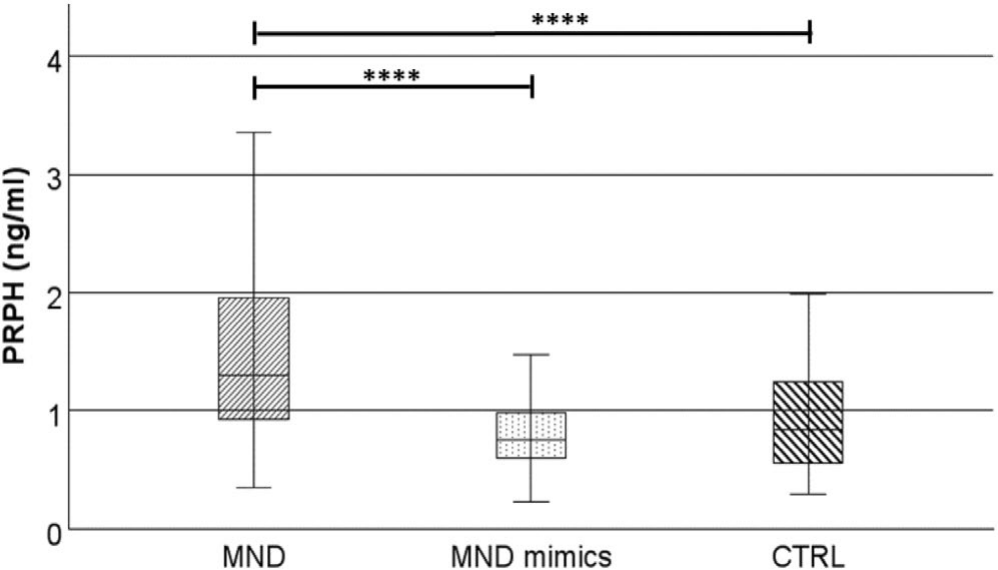
Levels of plasma PRPH in MND, MND‐mimics, and HCs. HCs, healthy controls; MND, motor neuron disease; PRPH, Peripherin; ****p ≤ 0.0001. Box plots show median and interquartile range.

Plasma PRPH levels were significantly different (Kruskal–Wallis: chi‐squared = 70.4, df = 2, *p* = 5.55 × 10^−16^; Figure 1) in MND patients (1.49 ± 0.63 ng/mL), MND mimics (0.79 ± 0.34 ng/mL), and HCs (0.94 ± 0.44 ng/mL). Post hoc Bonferroni's correction revealed elevated PRPH in MND patients compared to MND mimics (*p* = 5.33 × 10^−15^) and to HCs (*p* = 2.55 × 10^−6^). These differences are still more pronounced if we exclude PLS and PMA and we consider only ALS (PRPH levels 1.53 ± 0.64 ng/mL; ALS vs. MND mimics: chi‐squared = 70.6, df = 2, *p* = 4.44 × 10^−16^). When stratified by MND subtype (ALS, PLS, PMA), plasma PRPH concentrations were marginally lower in PLS and PMA (1.26 ± 0.45 ng/mL) than in ALS (1.53 ± 0.64 ng/mL); however, robust statistical comparison was not feasible because only four PMA cases were available. No differences between ALS/FTD and the other ALS patients (Mann–Whitney, *p* = 0.483).

Based on these findings, we calculated the ROC curve for PRPH to differentiate ALS patients and MND mimics, finding an AUC of 0.846 (IC 0.791–0.902) and the best cut‐off level of PRPH at 1.06 ng/mL with a Youden index of 0.54, with a sensitivity of 0.770 and a specificity of 0.767 (Figure S5).

Moreover, we looked at the 15 PLS and at the 12 HSP included in our study, two overlap diseases with a similar onset and similar clinical characteristics during their course not easy to discriminate from one another without the genetic test. We observed that PRPH levels were significantly higher (Mann Whitney, *p* = 7.71 × 10^−6^) in PLS (1.35 ± 0.49 ng/mL) than in HSP (0.48 ± 0.25 ng/mL).

Within the ALS subgroup, patients harboring mutations in one of the four major known genes did not exhibit different levels of PRPH compared to those without genetic mutations (*p* > 0.05).

No statistically significant differences in PRPH plasma levels were identified between ALS cases with bulbar and spinal onset (Mann–Whitney, *p* = 0.91) nor among ALS with different phenotype [2] nor between male and female patients (Mann–Whitney, *p* = 0.98).

### 3.3 Correlation of Plasma PRPH Levels With Clinical, Laboratory, and Instrumental Parameters in ALS Patients

**FIGURE 2:**
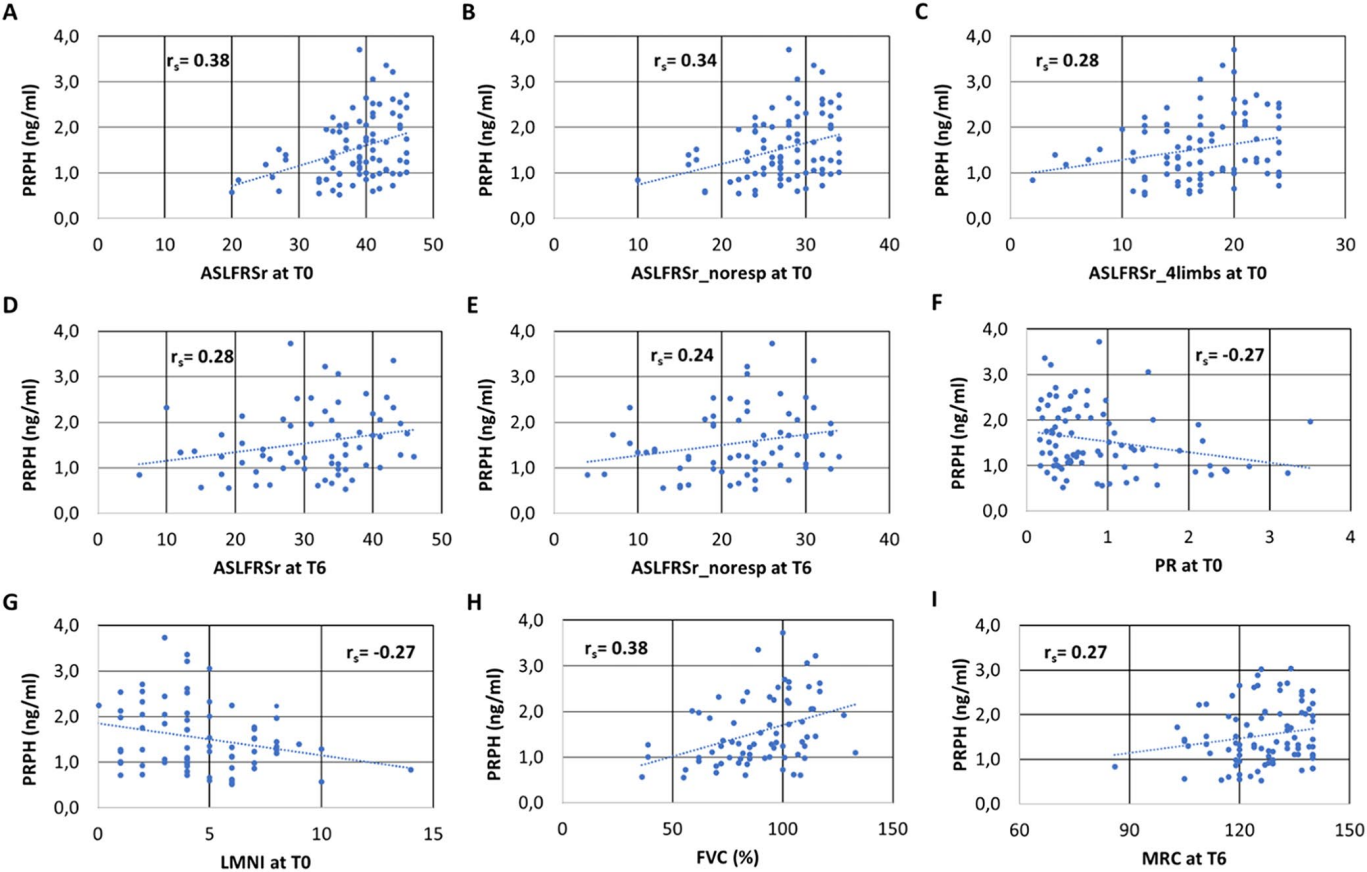
Correlation between clinical parameters and PRPH in ALS patients. ALFRSr_4limb at T0, Ssum of item 4,5,6,7,8,9 of the ALSFRSr at baseline; ALSFRSr at T0, ALS Functional Rating Scale‐revised total score at baseline; ALSFRSr at T6, ALS Functional Rating Scale‐revised total score at month 6; ALSFRSr_noresp at T0, ALSFRSr without the respiratory item at baseline; ALSFRSr_noresp at T6, ALSFRSr without the respiratory item at month 6; FVC, Forced Vital Capacity; LMNI, Lower Motor Neuron Index; MiToS, Milano‐Torino staging system; PRPH, Peripherin.

To reduce clinical heterogeneity, analyses focused on ALS patients. A modest positive correlation was noted between plasma PRPH levels and ALSFRSr at T0 (*r*_*s*_ = 0.364; *p* = 0.0002; Figure 2A), as well as ALSFRSr_noresp at T0 (*r*_*s*_ = 0.326; *p* = 0.0009; Figure 2B), ALSFRSr_4limbs at T0 (*r*_*s*_ = 0.261; *p* = 0.009; Figure 2C), ALSFRSr at T6 (*r*_*s*_ = 0.294; *p* = 0.015; Figure 2D), ALSFRSr_noresp at T6 (*r*_*s*_ = 0.257; *p* = 0.035; Figure 2E), and MRC at T6 (*r*_*s*_ = 0.266; *p* = 0.034, Figure 2I), whereas a negative correlation was observed between PRPH levels and PR at T0 (*r*_*s*_ = −0.225; *p* = 0.02; Figure 2F), and with LMNI at T0 (*r*_*s*_ = −0.255; *p* = 0.024; Figure 2G). Moreover, baseline PRPH predicted six‐month change in progression, calculated as the changes in progression rate (ΔPR%): a moderate inverse correlation was observed between PRPH at T0 and ΔPR% (*r*_*s*_ = −0.343, *p* = 0.006). All relationships remained significant after adjustment for sex, age, and site of onset (Table S5).

In support of the above‐reported evidences, PRPH was greatest in early phases of disease, measured using the established MiToS and KINGS staging systems (ordinal logistic regression analysis: MiToS, Chi‐square = 7.897, *p* = 0.005; KINGS, Chi‐square = 5.897, *p* = 0.015). Multivariable models covarying for age, sex, and site of onset strengthened these associations (MiToS, Chi‐square = 15.303, p_model = 0.004, p_PRPH = 0.01; KINGS, Chi‐square = 15.936, p_model = 0.003, p_PRPH = 0.026).

In a subset of 74 ALS patients with contemporaneous availability of forced vital capacity (FVC) collected around the data of blood sampling, PRPH positively correlated with FVC (*r*_*s*_ = 0.382; *p* = 0.0008; Figure 2H). This result is confirmed after multiple regression analysis covarying for ALSFRSr at T0, sex, and age (Table S5).

In contrast, no correlation was observed at either baseline or at six‐month follow‐up with PRPH levels and age at blood sampling, MRC score, change in MRC score (ΔMRC), UMNBS, and disease duration to sampling.

### 3.4 Survival Analysis

**FIGURE 3:**
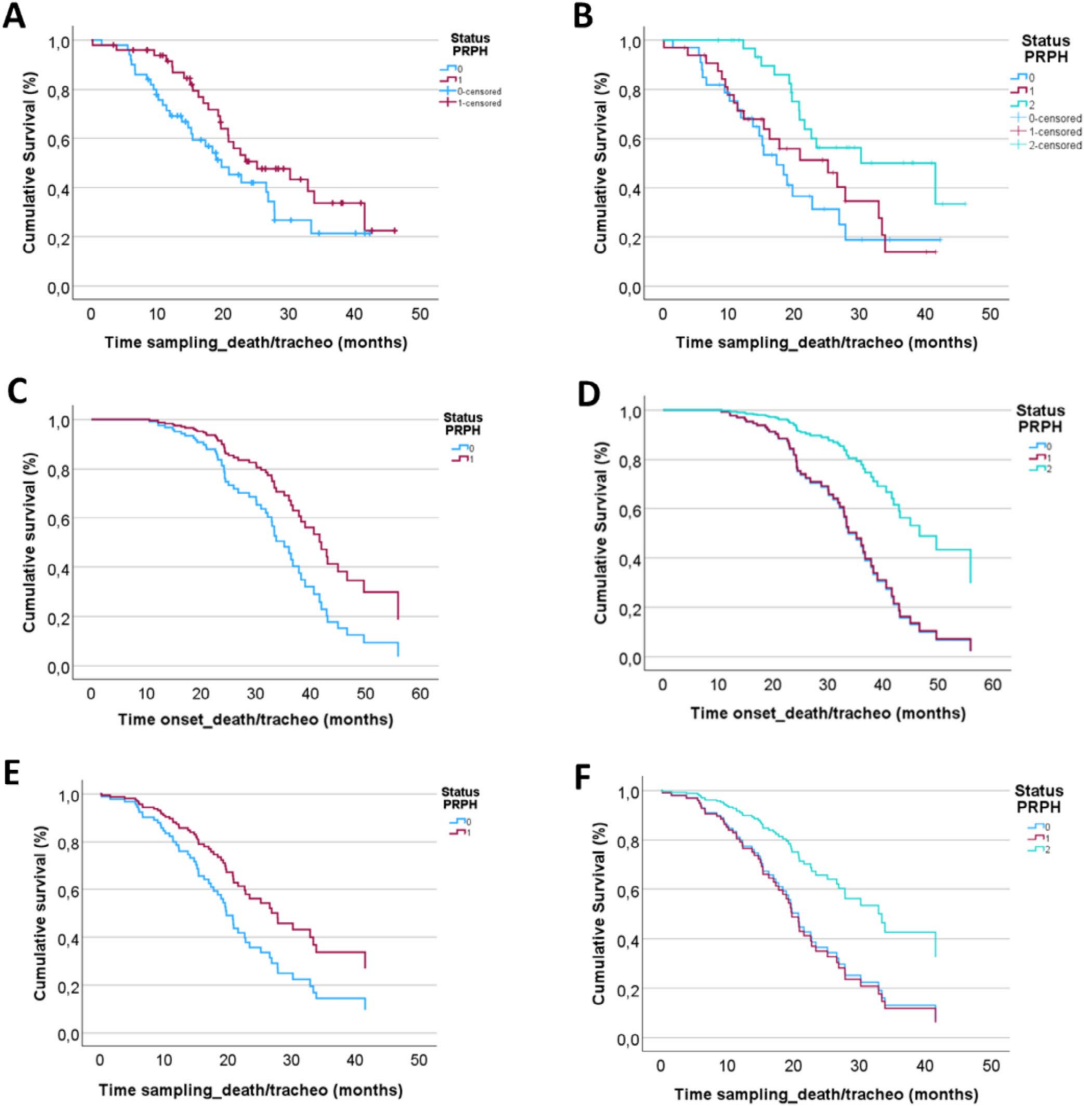
—Survival analysis. (A) and (B) represent the Kaplan–Meier analysis, respectively categorizing plasmatic PRPH levels using median and tertiles. (C) and (D) show survival curves covarying for plasmatic PRPH levels, sex, age at blood sampling, site of onset of the disease, and time from onset to death/tracheostomy, respectively categorizing plasmatic PRPH levels using median and tertiles. (E) and (F) show survival curves covarying for plasmatic PRPH levels, sex, age at blood sampling, site of onset of the disease, and time from blood sampling to death/tracheostomy, respectively categorizing plasmatic PRPH levels using median and tertiles. In (A), (C), and (E) the light‐blue line (status PRPH 0) corresponds to levels of PRPH below 1.35 ng/mL, whereas the red line (status PRPH 1) to levels of PRPH above 1.35 ng/mL; in (B), (D), and (E) the light‐blue line (status PRPH 0) corresponds to the 1° tertile (0.52–1.17 ng/mL), the red line (status PRPH 1) to levels of PRPH in the 2° tertile (1.19–1.72 ng/mL), and the light‐green line (status PRPH 2) to levels of PRPH in the 3° tertile (1.75–3.03 ng/mL). Patients with higher levels of PRPH show a longer survival.

Among the 100 ALS patients enrolled, 56 had either deceased or undergone tracheostomy by February 2024. Interim survival analyses were performed within this subgroup, acknowledging the limited sample size. Given the observed correlation between plasma PRPH levels, functional impairment, and disease progression, Kaplan–Meier survival curves were generated. PRPH levels were categorised using both the median value (≤ 1.35 ng/mL vs. > 1.35 ng/mL) and tertiles (1st: 0.52–1.17 ng/mL; 2nd: 1.19–1.72 ng/mL; 3rd: 1.75–3.03 ng/mL), with tertile stratification demonstrating superior representation due to the log‐normal distribution of PRPH data (Figure S6). Using the median‐based stratification, a borderline statistically significant divergence in survival curves was observed (Log‐Rank test, *χ* ^2^ = 2.731, *p* = 0.096; Breslow test, *χ* ^2^ = 4.420, *p* = 0.036; Tarone–Ware test, *χ* ^2^ = 3.803, *p* = 0.050, Figure 3A), which became more pronounced when stratified by site of onset (Log‐Rank test, *χ* ^2^ = 4.063, *p* = 0.044; Breslow test, *χ* ^2^ = 5.138, *p* = 0.023; Tarone–Ware test, *χ* ^2^ = 5.113, *p* = 0.024). Tertile stratification revealed a significant increase in survival in patients with higher PRPH plasma levels (Log‐Rank test, *χ* ^2^ = 9.088, *p* = 0.011; Breslow test, *χ* ^2^ = 11.128, *p* = 0.004; Tarone‐Ware test, *χ* ^2^ = 10.551, *p* = 0.005; Figure 3B). This significance persisted when further stratified by site of onset (Log‐Rank test, *χ* ^2^ = 12.812, *p* = 0.002; Breslow test, *χ* ^2^ = 13.981, *p* < 0.001; Tarone–Ware test, *χ* ^2^ = 14.341, *p* < 0.001).

Univariate and multivariate Cox regression analyses were conducted for both continuous and categorical PRPH values at the time of blood sampling (Table S6). Elevated plasma PRPH levels were consistently associated with improved survival, particularly when using tertile stratification. These findings were confirmed for overall survival and survival post‐blood sampling, using both continuous and categorical PRPH values. Graphical representations from multivariate Cox regression analyses both categorizing for median and for tertiles are displayed below in Figure 3C–F.

### 3.5 Longitudinal Evaluation

**FIGURE 4:**
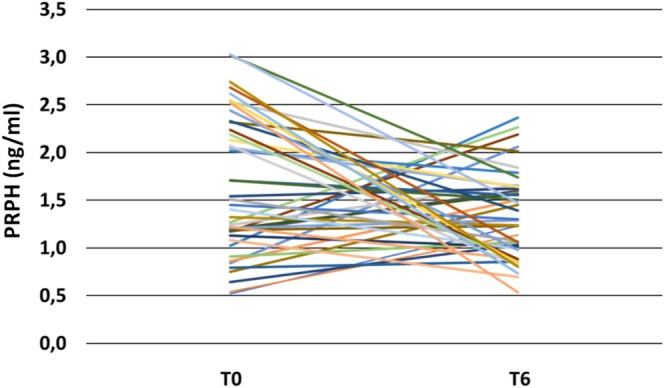
Longitudinal changing of plasma PRPH. PRPH, Peripherin; ΔPRPH%, percentage of change in PRPH levels between T0 and T6.

No differences (Mann–Whitney *p* > 0.1) are observed at baseline in terms of gender (M:F ratio 0.58 vs. 0.54), age (64.5 ± 11.9 vs. 64.1 ± 9.5), ALSFRSr (38.8 ± 4.8 vs. 38.3 ± 5.7), PR (0.90 ± 0.93 vs. 0.96 ± 0.98), MRC (126 ± 11 vs. 127 ± 11), and FVC (89 ± 20 vs. 90 ± 20) between the general group of 100 ALS patients and the subgroup of 41 subjects with ALS, for whom we measured PRPH levels approximately 6 months (mean: 6.68 ± 1.67; median 6.37 [IQR 5.30–7.73]) after the initial collection. Major clinical parameters at T0 and at T6 of these 41 patients are shown in Table S7. Analyzing the longitudinal trend of plasma PRPH levels, no statistically significant variations are observed between T0 and T6 (Wilcoxon test, *p* = 0.052), although the trend is decreasing (T0 1.63 ± 0.73 ng/mL; T6 1.33 ± 0.46 ng/mL; Figure 4). A moderate inverse correlation was observed between the percentage change in PRPH levels and the percentage change in PR values between T0 and T6 (*r*_*s*_ = −0.452, *p* = 0.008; Figure S7).

## 4 Discussion

This is the first comprehensive longitudinal study of plasma PRPH as a diagnostic and prognostic biomarker across the MND spectrum disorders. Plasma PRPH distinguished MND, particularly ALS, from mimic disorders with good accuracy, identifying an optimal cutoff of 1.06 ng/mL. Furthermore, as detailed below, our findings suggest that plasma PRPH may reflect compensatory responses to motorneurone injury. These data corroborate earlier serum findings [11] in a larger longitudinal cohort and, crucially, demonstrate the utility of plasma PRPH, a biofluid previously unexamined.

Drawing from these observations, the potential diagnostic utility of PRPH in patients in whom ALS is suspected is conceivable. Nevertheless, broader confirmation, including a concomitant comparison with other established neurofilaments, paralleled by adherence to standard laboratory techniques, remains essential, as reported in a previous study [12].

Another promising application of PRPH lies in distinguishing PLS from HSP before genetic testing, which is time‐consuming and costly. Although these findings require larger multicenter validation, PRPH could improve early diagnostic efforts in pure upper motor neuron diseases.

Analysing the absolute levels of PRPH, we observed a substantial variability among the three available studies [11, 12], including the present one. These discrepancies may stem from variations in laboratory techniques, different kits employed, variances in sample processing methodologies, different atmospheric conditions, different dilutions, and distinctions in the type of material employed (plasma versus serum). Speculation may also extend to a potential blood matrix effect resulting in the sequestration of PRPH in aggregates or its degradation by proteases or immunological binding, consequently reducing the free fraction, akin to what happens in plasma for NfL and pNfH [25].

Focusing on ALS, plasma PRPH levels showed no associations with genetic factors, gender, age, ALS phenotype, disease site of onset, cognitive impairment, UMNS, and common blood examinations. In contrast to the findings reported in another study [11], modest correlations were noted with clinical parameters. Specifically, higher PRPH levels correlated with less severe disease at the time of sampling (per staging systems) and better functional status (ALSFRSr, including sub‐scores, and MRC). Furthermore, elevated plasma PRPH levels were associated with a lower disease progression rate prior to blood sampling and appear to predict a reduction in progression rate in the following months, independent of disease stage. Additionally, PRPH was inversely correlated with lower motor neuron damage both at the time of sampling and after 6 months, as assessed by the LMNI score aligning with its physiological concentration in the peripheral nervous system. An intriguing correlation between PRPH levels and FVC suggests that ALS patients with better‐preserved respiratory function exhibit higher PRPH levels.

Survival analyses further supported PRPH's prognostic value, showing that ALS patients with higher baseline PRPH levels had longer survival times, as evidenced by the graphs concerning both the time elapsed between onset and death/tracheostomy and that between sampling and death/tracheostomy. However, ongoing follow‐up (nowadays, fortunately, 44 patients with ALS out of 100 are alive) and validation in larger multicentre studies are needed to solidify these findings.

All the above‐mentioned data suggest that, unlike neurofilaments, which directly correlate with disease progression, the number of regions affected, and motor neuron damage [26, 27, 28], plasma PRPH levels may not indicate the extent of motor neuron impairment. Instead, PRPH levels could be an index of the intracellular repair attempts initiated by the motor neurons early in the disease. Elevated PRPH in early stages and its inverse relationship with disease progression contrast with typical neurodegenerative biomarkers. If this hypothesis holds true, PRPH levels could potentially increase as part of an upregulation process in response to neuronal injury, a phenomenon already observed in different cellular studies [29, 30, 31]. In particular, an elevation of PRPH blood levels has been described both in lesional models of the lower motor neuron and in genetic models of lower motor neuron neurodegeneration, and the level of PRPH correlates with the entity of lower motor neuron effort of biological repair.

Furthermore, the higher levels of PRPH in SBMA patients compared to ALS observed in another study [11] support the idea of PRPH not merely as a marker of damage but as an indicator of mechanisms mitigating lower motor neuron damage. In fact, the less aggressive nature of SBMA, with slower progression rates, may explain this difference. Unfortunately, based on this study, we cannot determine the directionality of the causal effect: whether peripherin increases due to its involvement in the supposed repair attempt or its increase is merely an epiphenomenon of this process.

To clarify, when we talk about the attempt of repair and the mitigation of lower motor neuron damage, we do not mean a real clinical improvement of the disease, which is for definition relentlessly progressive, but merely we concern ourselves with a cellular compensatory mechanism. Future investigation of these mechanisms could open the field to a better understanding of rescue attempts of motor neurons in ALS and could lead to identifying possible targets of therapies.

However, such interpretations remain speculative: comprehensive and multicentre studies with extended follow‐up periods, along with histological and neuropathological data and with confirmation in cellular and animal models, are necessary to validate these hypotheses.

The limitations of our study encompass the absence of additional fluid biomarkers concurrently measured (such as NfL and pNfH), the absence of ultrasensitive techniques, such as single‐molecule array technology (SiMoA), the lack of validation evidence in preclinical models of ALS, the monocentric design of the study, and challenges in comparing these findings with the other two studies due to the utilization of plasma instead of serum in absence of a validated and standard protocol.

In conclusion, the novelty of our study and its contribution to the existing literature consist in defining PRPH useful for the early differential diagnosis of ALS, and in highlighting its prognostic role since the beginning of the disease. Our findings also imply that PRPH might be an index of endogenous motorneurone repair. Confirmation in larger cohorts, using next generation quantification technologies, is now warranted.

## Author Contributions

**Alessandro Bombaci:** conceptualization, software, methodology, data curation, formal analysis, writing – original draft, writing – review and editing, supervision. **Giovanni De Marco:** resources, writing – review and editing. **Federico Casale:** writing – review and editing, resources. **Paolina Salamone:** writing – review and editing. **Giulia Marchese:** writing – review and editing. **Giuseppe Fuda:** writing – review and editing. **Andrea Calvo:** writing – review and editing, supervision. **Adriano Chiò:** writing – review and editing, supervision, project administration, methodology.

## Ethics Statement

Ethical approval was granted by the Turin ALS Centre's Ethics Committee (Comitato Etico Azienda Ospedaliero‐Universitaria Città della Salute e della Scienza, Torino) (n° 0011613, 03/02/2020).

## Consent

All participants provided written informed consent.

## Conflicts of Interest

The authors declare no conflicts of interest.

## Supporting information


Data S1.

## Data Availability

The data that support the findings of this study are anonymized and openly available in Zenodo at https://doi.org/10.5281/zenodo.15301746.
